# The experiences, perspectives and needs of families raising neurodivergent twins: a multi-informant photo-elicitation exploratory study

**DOI:** 10.3389/fpsyg.2025.1571108

**Published:** 2025-06-03

**Authors:** Vicky Foley, Myrofora Kakoulidou, Vassilis Sideropoulos, Camilla Phelps, Georgia Pavlopoulou

**Affiliations:** ^1^Department of Psychology & Human Development, IOE, UCL’s Faculty of Education and Society, University College London, London, United Kingdom; ^2^Department of Primary Care and Population Health, Institute of Epidemiology and Health Care, University College London, London, United Kingdom; ^3^Group for Research in Relationships and NeuroDiversity (GRRAND), Department of Clinical, Education and Health Psychology, Division of Psychology & Language Sciences, Faculty of Brain Sciences, University College London, London, United Kingdom; ^4^Anna Freud, London, United Kingdom

**Keywords:** relationships, siblings, twins, family wellbeing, photo-elicitation, neurodiversity

## Abstract

Despite research suggesting the importance of secure sibling relationships and the uniqueness of twin siblings’ connections, little is known about the experiences of twin siblings who have a neurodivergent twin. This study sought the voices of the twin/triplet siblings about their first-hand experiences of living with a neurodivergent co-twin/triplet. Research questions were co-designed with four mothers of neurodivergent twins/triplets, after consulting with 38 families of neurodiverse twins about their top research priorities. Fifteen photo-elicitation semi-structured interviews were conducted to explore both the neurotypical twins’ experiences and perceived needs and the mothers’ perceptions of the neurotypical twins’ experiences and perceived needs. Interpretative Phenomenological Analysis (IPA) was used to analyse the interview data. Themes across the neurotypical twins and their mothers included: (i) perception of the relationship between twins, (ii) perception of neurotypical twins as experts of their sibling’s neurodivergence, (iii) perception of the neurotypical twin’s struggles and needs. This study illustrates the everyday experiences of neurotypical twins with a neurodivergent co-twin from multiple perspectives. The themes demonstrate the closeness of their twin bond but also depicts the struggles sharing attention and time. A key difference in their perspectives was that mothers were more worried about their twins’ differences and conflicts, while twins felt mutual understanding and normalised these differences despite their conflicts. Future research should further explore twins’ life experiences and needs across the lifespan and include the perspectives of all family members, including those of neurodivergent twins as well as dyads and triads where all twins are neurodivergent.

## 1 Introduction

Sibling relationships are among the most enduring and longest-lasting bonds in a person’s life. Typically close in age and beginning early in life, these relationships often span an individual’s lifetime (Milevsky and Heerwagen, 2013). Siblings play a vital role in child development, influencing cognitive, emotional, social, and linguistic growth. Over time, these relationships evolve in complexity. In early childhood, siblings often act as primary playmates, helping each other learn social rules and emotional regulation. Between the ages of 5 and 11, sibling interactions become more nuanced, involving greater emotional understanding, conflict resolution, and negotiation skills. The extensive contact and companionship that siblings share in childhood provides ample opportunity for mutual influence on socioemotional development and adjustment (Pike et al., 2005).

Research shows that siblings who perceive strong social support from each other tend to report higher self-esteem and life satisfaction, along with lower levels of depression and loneliness, compared to those with minimal sibling support (LeBouef and Dworkin, 2021; Milevsky, 2005). Longitudinal studies further suggest that sibling affection can buffer the negative effects of stressful life events by mitigating internalising behavioural issues (Gass et al., 2007; Kramer, 2010).

In the UK, approximately one in every 65 pregnancies results in a multiple birth (Twins Trust, 2019). The use of in-vitro fertilisation has significantly increased the incidence of multiple births (Malizia et al., 2013), yet the literature on the lived experiences of twins and other multiples—whether neurotypical or neurodivergent—remains limited. Although the rate of multiple births has risen over recent decades (Monden et al., 2021), research into the dynamics of twin relationships has not kept pace. As a result, key questions concerning the nature, characteristics, and developmental trajectories of these relationships remain unanswered. This gap leaves parents, educators, and clinicians without essential insights to support and enhance these unique sibling bonds.

Research has shown that twin relationships, particularly among monozygotic (MZ) twins—who share nearly 100% of their genetic material—are often marked by strong closeness and interdependence in adulthood (Fraley and Tancredy, 2012). However, it is unclear whether these traits are also present during childhood. From conception, twins spend significantly more time together than other sibling dyads and often report higher levels of closeness (Bekkhus et al., 2014). Levels of closeness can also vary by zygosity, with MZ twins generally demonstrating greater emotional intimacy and more frequent contact (Fortuna et al., 2011). Tancredy and Fraley (2006) suggest that twins form particularly close bonds due to their shared environments and experiences, such as birthdays, friend groups, and daily routines.

In multiple births, the likelihood of developing a disability or form of neurodivergence is higher compared to singleton pregnancies (Human Fertilisation and Embryology Authority, 2022). Lorenz (2012) notes that twins tend to have lower birth weights and shorter gestational ages, which are associated with increased risk of disability. Additional factors also contribute to differences in neurodevelopmental milestone attainment between twins and singletons (Gnanendran et al., 2015; Smith et al., 2007; Wadhawan et al., 2009; Zeitlin et al., 2010). However, significant variation in study design, follow-up periods, and measurement tools complicates interpretation (Bodeau-Livinec et al., 2013; de Zeeuw et al., 2012; Eras et al., 2013; Gnanendran et al., 2015).

Neurodiversity refers to the concept that neurological differences are natural and should be recognised and respected as such (Pellicano and Houting, 2022). Common forms of neurodivergence include autism, attention deficit hyperactivity condition (ADHC, also known as ADHD), learning disabilities, Down syndrome, and cerebral palsy (Patel, 2011). The neurodiversity paradigm encourages acceptance and accommodation of these differences, rather than viewing them as deficits or disorders.

Parents of twins often strive to distribute their attention equally, but this can be challenging when one twin is neurodivergent and requires additional support, for instance, with feeding or dressing (Clark and Dickman, 1984). Historically, research on siblings of neurodivergent children has emphasised a deficit or tragedy narrative, highlighting risks and adjustment challenges while overlooking the enriching aspects of these relationships (Saxena and Adamsons, 2013). More recent studies have shifted towards more positive and accepting frameworks, highlighting the importance of lifespan support and broader societal change to improve quality of life (Hayden et al., 2019; Meltzer and Kramer, 2016; Pavlopoulou and Dimitriou, 2020). However, these studies have not focused on twins or triplets, leaving their specific perspectives and needs unaddressed.

Some families report positive experiences of having a neurodivergent sibling (Kovshoff et al., 2017), while others cite increased behavioural challenges, particularly among closely aged siblings (Dyson, 1989)—a dynamic with clear implications for twins. Little is known about how twins and their parents perceive the influence of neurodivergence or disability on twin relationships, especially when one twin is neurotypical.

This qualitative interview study explores the lived experiences—social, emotional, and practical—of neurotypical twins aged 5–11 who have a neurodivergent twin sibling, along with the perspectives of their mothers. A qualitative methodology was chosen for three primary reasons. First, most twin/triplet research originates in behavioural paediatrics, focusing on developmental risks through long-term behavioural tracking and medical records. For instance, twin pairs—whether MZ (identical) or DZ (fraternal)—have been used to explore genetic and environmental influences on traits (McGuire, 2001). This body of work has largely centred on developmental distortions and health-related issues, such as behavioural problems (Trouton et al., 2002), health risks (Aarnio et al., 2002), substance use (Kujala et al., 2007; Eaves et al., 1999; Penninkilampi-Kerola et al., 2006), and addiction (LaBuda et al., 1997). However, these studies focus on sibling similarities and differences, rather than the experiences and needs of neurotypical twins themselves (McGuire et al., 2015).

Second, much of the existing research relies on maternal reports to assess sibling experiences (Griffith et al., 2014; Meyer et al., 2011; Petalas et al., 2012). Hastings and Petalas (2014) found discrepancies between maternal accounts and sibling self-reports, highlighting the need to include both perspectives for a more valid understanding. Even when twins are part of the sample (e.g., Hastings, 2007), their specific experiences are rarely reported. Some studies focus on maternal experiences of raising twins (Bolch et al., 2012; De Vos et al., 2002) or use maternal reports to examine how neurotypical twins experience growing up with a neurodivergent co-twin (e.g., McDougall et al., 2006, in the context of ADHD).

Third, much of what is known about twin relationships during childhood is based on retrospective accounts (e.g., Määttä et al., 2016), rather than direct testimony from children themselves. Only one study was identified that directly investigated the experiences of neurotypical twins aged 18–25 who grew up with a twin with profound intellectual and multiple disabilities (Niedbalski, 2024). Participants in that study described both enriching and challenging aspects of their relationships. Our qualitative interview approach aims to address this gap by exploring how neurotypical twins perceive their sibling relationship during childhood, and how mothers understand and support those relationships and the needs of their neurotypical twins.

We chose to focus on the experiences of children aged 5–11 because this period is developmentally significant, shaping early understandings of identity, disability, and belonging (Murray, 2000). Yet, research often excludes children in this age range, based on assumptions about their developmental capacity to articulate lived experiences (Crane and Broome, 2017). By foregrounding the voices of children and their mothers, this study offers a rare, developmentally attuned perspective on the sibling dynamics of neurotypical twins growing up with neurodivergent co-twins.

### 1.1 Community involvement

The impetus for this study was the growing number of parents who asked the corresponding author for more attention on understanding the experiences and needs of parents and their twins/triplets during a community event back in 2018. This was after a talk on siblings’ relationships when at least one child is neurodivergent.

Following the example of Pavlopoulou et al. (2022), we ran a series of community-based consultations to establish the focus of this study. This was initiated by four mothers with lived experience (one is also a co-author in this paper). One of them was a twin herself and a sibling of a neurodivergent brother. We collaboratively developed a survey intended for all parents who were members of a closed national social network group with parents of twins and triplets in England. The survey allowed parents of twins and triplets to share their research priorities, detailing which areas they would like our research team to look at; however, the responses were exclusively from mothers of twins. 38 mothers completed a self-administered co-produced survey online (see Supplementary material 1 for survey questions). The results showed that they rated twins’ experiences and relationships as the most important priority, with 76% of respondents deeming this vitally important. The next priority was to look at marital relationships, with 66% of respondents deeming this vitally important. The next priorities were sleep problems (55%), school provision, homework and after school activities (53%) and social problems and stigma in everyday life (34%).

In collaboration with the last author, the four mothers also discussed pre-study considerations for this study, participant recruitment, data collection considerations, and post-study considerations to build equal partnerships with participants during the advisory process following the Gowen et al. (2019) guidelines. This paper reports on a preliminary examination of one of the top research priorities, which is the everyday family relationships and experiences of twins.

### 1.2 This study

Despite research suggesting the importance of sibling relationships and the uniqueness of twin sibling’s connections, little is known about the experiences of twin siblings who have a neurodivergent twin. This study elicited the voices of neurotypical twin siblings who have first-hand experiences as to what it is like to live with a neurodivergent co-twin. In addition, the mothers of the twins shared their perspectives. Due to the lack of research concerning the experiences of neurotypical twin siblings living with a neurodivergent co-twin, the proposed research question for this study is: what are the experiences, perspectives and needs in families raising neurodivergent twins? The priorities identified via the survey and the consensus collaborative group we worked with influenced the aims of the study, as described below

1. To understand the siblinghood experiences and perceived needs of neurotypical twins who have a neurodivergent co-twin.
2. To understand the mother’s perspective of the siblinghood experiences and perceived needs of neurotypical twins who have a neurodivergent co-twin.

## 2 Method

### 2.1 Design

The research used a qualitative design. Background data were obtained through a questionnaire which explored the mothers’ experiences of raising a neurodivergent child looking at the parents’ understanding of their child’s neurodivergence and their quality of life (see Supplementary material 2). Qualitative data were obtained from the neurotypical twins and their mothers using a photo-elicitation interview. The interviews explored their perspectives on the experiences and needs of the neurotypical twins growing up in a family with a neurodivergent twin (see Supplementary material 3 for the interview schedules for twins and mothers).

### 2.2 Participants

Thirteen participants of which six neurotypical twin siblings with a neurodivergent twin brother and seven mothers (of the twins) participated in the interviews. One mother participated without their neurotypical child. All mothers also completed some background questionnaires.

Opt-in purposive sampling was used for participant recruitment. The primary criteria for eligibility were: (1) participants were twins/triplets (monozygotic or dizygotic) one of whom was neurodivergent between 5 and 11 years old or their parents and (2) the twins/triplets had to be living together. Participants were recruited through online charities specialising in neurodivergent children.

The mean age of the twins was 8.43 years (SD = 2.07). All the twins were monozygotic, 57% were female twin siblings and 43% were male twin siblings. Mothers’ age ranged from 43 to 55 years (mean = 47.86 years, SD = 4.56). The types of neurodivergence ranged from autism (42.85%), cerebral palsy (28.5%), a rare chromosome condition (14.3%) and epilepsy and cerebral palsy (14.3%). All the neurotypical twins were aware of their twins’ siblings’ neurodivergence. Table 1 shows the participant demographics. All quantitative data were analysed on SPSS.

**Table 1:** Participant demographics.

Pseudonym for neurotypical child/mother	Twins/triplets age in years	Neurotypical twin’s gender	Neurodivergence of the twin	Marital status of mother	Ethnicity	N of family members
Emmanuel/Anne	6.5	Male	Rare chromosome condition—dup 2q32.3-q33.3, 9q31.2	Married	White	4
Connie/Joshua	7	Male	Autism, epilepsy, hydrocephalus, CP, moderate sensory-neuro deafness, learning disability	Married	White	4
^*^/Louise	7	Female	Cerebral palsy (mixed triplets, two male triplets have this diagnosis)	Married	White	6
Amelia/Julia	9	Female	Autism	Married	White	4
Orla/Jill	8	Female	Quadriplegic Spastic Cerebral Palsy	Married	White	5
Liam/Kelly	12	Male	Autism, ADHD and a microdeletion of chromosome 16p11.2	Married	White	5
Reya/Amena	10	Female	Autism	Separated	Asian	5

* Neurotypical child did not wish to take part in the interview.

### 2.3 Measures

#### 2.3.1 Background questionnaires

An online questionnaire was co-created and sent to the mothers to complete in their own time. In addition to the demographic information summarised above, two additional measures were included in the questionnaire (see Supplementary material 2 for the questionnaires for mothers indicating background data to help reader understand profile of mothers).

The mothers’ knowledge and beliefs of their neurotypical child’s were measured using the Sheffield Learning Disability Outcome Measure (SLDOM; Girgis, Sheffield Children’s NHS Foundation Trust, and CORC, 2013). The SLDOM is a validated parent-report tool designed to assess functional outcomes across key domains of daily living for children with neurodevelopmental conditions. These domains include communication, independence, socialisation and emotional regulation. The SLDOM uses a Likert scale to capture the level of difficulty a child experiences in each domain, with higher scores reflecting greater functional challenges. The purpose of including the SLDOM in this study was to gain a structured understanding of the functional profile of the child, as perceived by the parent. In the SLDOM, a parent’s ‘understanding’ of their child’s neurodivergence refers to the depth of insight they possess into how their child’s neurodevelopmental profile affects day-to-day functioning, emotional experiences, and social interactions. This includes awareness of their child’s strengths and difficulties, the ability to recognise sensory or behavioural triggers and a capacity to advocate for appropriate support. Such understanding is often shaped by personal experience, engagement with professionals and the parent’s own neurodevelopmental perspectives.

The SLDOM includes seven questions with responses given on a 5-point Likert scale, which ranges from ‘strongly agree’ to ‘strongly disagree’. A higher score indicates more affirming or supportive beliefs about the child’s neurodivergent identity. The items relate to the parents’ understanding of the child’s behaviour and their ability to cope with their child’s behaviour. The SLDOM is used routinely across children’s learning disability services although there is no evidence of psychometric reliability or validity yet. Delahunty et al. (2018) reported that it demonstrates good face validity and is a useful tool for providing information about the parent–child relationship when the child has a learning disability.

The quality of life of the mother was measured using the World Health Organisation Quality of Life questionnaire (World Health Organization, 1996). The WHOQOL_BREF is a 26-item questionnaire which contains questions from the WHOQOL-100 questionnaire. The responses are given on a 5-point Likert response scale, with higher scores indicating better quality of life. The WHOQOL_BREF has been used in previous research looking at the quality of life of parents of children with learning difficulties (Dardas and Ahmad, 2014; Shu, 2009) and has been found to demonstrate good psychometric properties including high internal consistency (alpha = 0.93) and construct validity (McConachie et al., 2018).

### 2.4 Photo elicitation interview

Photo elicitation interviews involve participants taking photographs and then discussing these photos during an interview (Mandleco, 2013). This method has been previously used with children to elicit richer qualitative accounts and to generate discussion into children’s lives accommodating preferred speaking or non-speaking ways of communication (Einarsdottir, 2005; Pavlopoulou and Dimitriou, 2020). The children typically receive a set of open-ended questions before the interview and the interview is led by them, as children bring photos to talk about the topics and issues that matter to them. This is an effective method of obtaining qualitative data and has been used with siblings of autistic children (Latta et al., 2013; Pavlopoulou and Dimitriou, 2020), Down syndrome (Rampton et al., 2007) and parents raising disabled children (Lassetter et al., 2007). In our interviews, we used photo-elicitation with both children and their mothers.

### 2.5 Ethics and procedure

This study received ethics approval by the UCL’s Department of Psychology and Human Development ethics committee. Recruitment occurred through online adverts and letters to specialist twin charities.

Participants followed a secure online link to give consent for taking part. Both mothers and neurotypical twin siblings offered written consent before taking part in the interviews.

After this, mothers completed an online background questionnaire. The questionnaire was divided into three parts: (1) background information on their family, (2) parents’ understanding of their child’s neurodivergence, and (3) parents’ quality of life. Mothers and twin siblings were interviewed separately to allow greater time and independence for discussions.

The first author contacted participants to verify their willingness to participate and to arrange the first home visit. Participants who gave consent to the photo-elicitation interviews were then given an ethics training session which explained the ethics around taking photos and outlined the criteria for the photos (see Supplementary material 4 for the ethics forms and photo training session materials). The mother and child were asked to take photos over the course of 1 week using their own equipment each. The neurotypical twin was asked to take photos which represent their thoughts, feelings, and experiences on what it’s like to have a twin with a disability and their sibling relationship. The mother was asked to take photos which represent their perspectives on the twin’s sibling relationship. As part of the ethics training, the mother was asked to review the selected photos with the child to make sure the child has consent for the photos they included and there was no nudity or other inappropriate material.

During the second home visit, individual interviews were held with the child and the mother. If the mother and child agreed to the photo interview, participants were asked to describe each photo, in the order, they were taken. Prompts were given for example ‘*can you tell me why you chose to take this photo?*’ and ‘*how did you feel when you took this photo?*’

A debrief was given to all participants at the end of the session. This included summarising the aims of the study, offering opportunities for questions and signposting to relevant support services. All the interviews were voice-recorded and transcribed verbatim.

### 2.6 Interview analysis and reflexivity

The data were analysed using interpretative phenomenological analysis (IPA). The aim of IPA is to explore people’s experiences and ways of viewing the world (Barker et al., 2002). In this study, IPA was used to capture the experiences of growing up with a neurodivergent twin as it is understood by the neurotypical twin and their mother.

The interviews were analysed manually by the core analysis team (FV GP). These were analysed using IPA based on the seven steps recommended by Smith et al. (2021). Audios of the interviews were transcribed manually line-by-line. First, the researchers familiarised themselves by reading and re-reading the first interview transcript (Step 1). The transcript was read several times, making comments on anything that appeared to be significant and any generated themes. Then, they made notes on anything of interest in the transcript (Step 2). The researchers developed personal experiential themes per participant (Step 3). They looked for connections between these themes and created tables clustering them together per participant (Step 4). In the next phase, the second interview was analysed and a list of personal experiential themes per participant was created (Step 5). This continued until all transcripts were analysed separately. Finally, the researchers looked for patterns across all transcripts (Step 6). They reflected on the personal experiential themes and generated a list of group experiential themes across all participants per group. The interviews of the neurotypical twins and their mothers were analysed separately. The results were not organised according to the research questions but based on the themes emerging from each group.

After VF reviewed two of the transcripts, she invited GP to conduct an independent analysis of the same transcripts to enhance the reliability of the findings and achieve agreement on the identified themes. In line with the approach suggested by Rodham et al. (2013), the researchers involved in the analysis listened to the audio recordings and engaged in discussions about the coding process to minimise the risk of imposing their own assumptions or biases on the data. Maintaining a curious mindset and actively practicing reflexivity were essential for this process.

Reflexivity is an integral and necessary part in phenomenological research (Engward and Goldspink, 2020). The self that one brings in research, including their multiple identities (e.g., disability, gender, ethnicity, race, socioeconomic status, age etc.) can influence the generation and interpretation of findings. We embedded reflexivity within each analysis step, with all the members of the core analysis team reflecting on their analysis approaches. The team had regular meetings to reflect on their positionality and discuss how their different backgrounds and expertise influenced the data analysis. This included the different expertise they brought into research and previous work with young people and families. For instance, as white females with varied professional, ethnic/cultural, academic and class backgrounds we discussed how these have shaped our siblinghood experiences as well as pre-conceptions we have about twins’ relationships and twins’ family experience.

In our literature review, we observed that discussions surrounding twins and their families are frequently approached from a professional standpoint. For example, there are medicalised examinations of twins prevalence, ability/disability etc. Our research seeks to emphasise the viewpoints of twins and their mothers.

The individuals we interviewed are not viewed simply as passive subjects within a set of objective, natural mechanisms. Rather, we acknowledge them as meaning-making agents whose lived experiences and perspectives shape the research process. Our goal is not to uncover the conditions that drive human behaviour. Instead, we focus on the everyday interactions and the meanings that arise from the experiences of their families. Additionally, we aim to explore diverse perspectives, recognising the inherently interpersonal nature of our world. It is essential to consider the significance of others and our relationships with them. The study adopts an experience-based collaborative and flexible approach to understanding family members’ experiences grounded in the lifeworld theoretical model of Galvin and Todres (2013), modified by Pavlopoulou and Dimitriou (2019). The framework draws together eight dimensions of human experience, which are key to the siblings’ wellbeing (insiderness, agency, uniqueness, sense-making, personal journey, sense of place, embodiment and togetherness), placing a person at the centre of their own ‘lifeworld’ to acknowledge and validate them as the expert of their own experiences (McGreevy et al., 2024). This epistemological approach shifts our understanding of sibling wellbeing away from a deficit-focused, medical narrative and towards meaningful engagement with their troubles and joys in the family environment.

## 3 Results

### 3.1 Background behavioural data

Of the 11 mothers who met the sample criteria, seven completed the questionnaire. The Sheffield Learning Disability Outcome Measure (SLDOM) was conducted to analyse the mother’s knowledge and beliefs of their child’s neurodivergence. The advice from the SLDOM is that scores over 25 are considered positive. We found that four out of the seven mothers who took part in the questionnaire had a positive score, signalling that they felt that they understood their child’s neurodivergence. This shows that almost half of the mothers had difficulties understanding their child’s behaviour, were not hopeful about the future and did not have much confidence.

The World Health Organisation Quality of Life questionnaire (WHOQOL-BREF) assessed the quality of life of the mothers. The questionnaire had four domains, physical health, psychological, social relationships, and environment. The responses are calculated out of 100 points. For the physical health domain, mothers had a mean score of 58 (SD = 11.70). In the psychological domain, mothers had a mean score of 45.4 (SD = 11.90). In the social relationship’s domain, a mean score of 42.25 (SD = 9.90). In the environment domain, a mean score of 56.38 (SD = 12.43). Across the board, the mothers’ scores were average, with social relationships scoring the lowest, showing that their quality of life is neither excellent nor poor.

### 3.2 Photographs

In total, the children took 26 photographs and talked about all of them in the interviews. Content analysis generated two major categories (i) photos associated with the twin’s neurodivergence (46%) and (ii) photos showing a togetherness with their twin (42%). The final 12% (*n* = 3) of the photos depicted sibling conflict. The photos associated with the twins’ neurodivergence, were mainly around food/mealtimes (*n* = 6, 23%), issues associated with the sibling’s neurodivergence (sensory issues/meltdowns/hygiene, *n* = 4, 15%), caring for the twin (*n* = 1, 4%), and their mother caring for the twin (*n* = 1, 4%). The photos associated with the twin’s relationship were mainly about fooling around (*n* = 4, 15%), playing together (*n* = 4, 15%), family times (*n* = 1, 4%) and twins (*n* = 2, 8%).

In total, the mothers took 61 photographs and talked about all of them in their interviews. Content analysis identified two major categories, photos showing the twin relationship (45%) and photos showing time together as a family (28%). The final photos were associated with the twin’s neurodivergence (15%), sibling conflict (7%) and time alone without the twin (5%). The photos associated with the twin’s relationship were mainly around playing together (*n* = 12, 20%), helping their twin learn new skills (*n* = 6, 9%), looking out for twin (*n* = 4, 7%), time together as twins (*n* = 4, 7%) and wanting to be like their twin (*n* = 1, 2%).

### 3.3 IPA themes for the neurotypical twins

Analysis of the six transcripts from the neurotypical twins’ interviews resulted in the generation of four group experiential themes, each of which included a list of personal experiential themes (see Table 2).

**Table 2:** Neurotypical children’s group experiential themes, personal experiential themes and example photos from interviews.

Group experiential theme	Personal experiential theme	Example photo
“There is fun… and there is tough stuff too sometimes”: Relationship between twins	Enjoyment from being together Intensity of bond between twins Sibling conflict	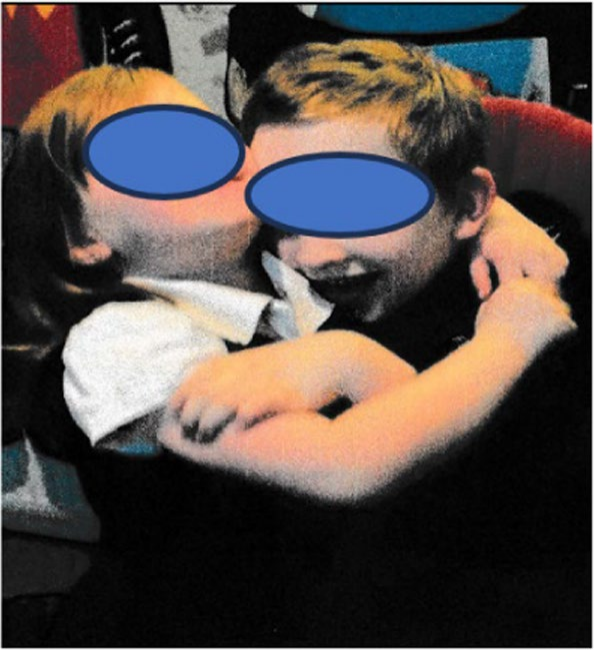
“…I get it, I know him so well.” Neurotypical twins as experts of their brothers’ neurodivergence	Perception of condition and behaviour Adapting and change	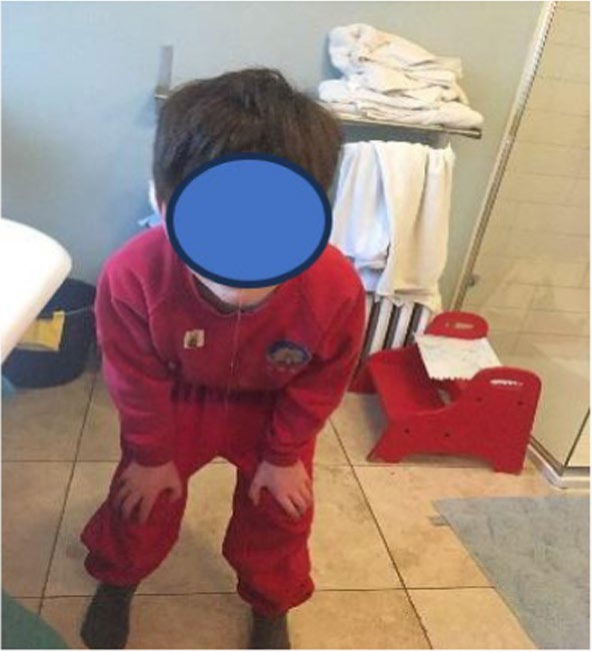
“It’s fun to be a twin but sometimes I feel worried.” Neurotypical twins’ perceived struggles and needs	Current worries/difficulties Perceived support	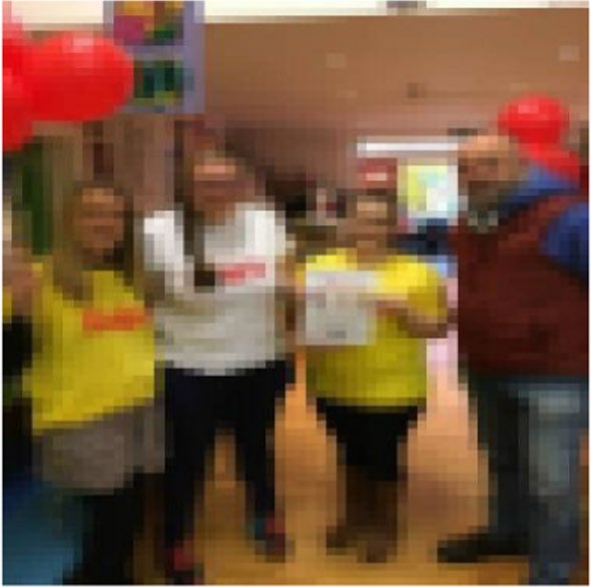
“My mum spends a lot of time with him, helping him.” Neurotypical twins’ relationship with parents	Differentiated parenting Worries about parental health	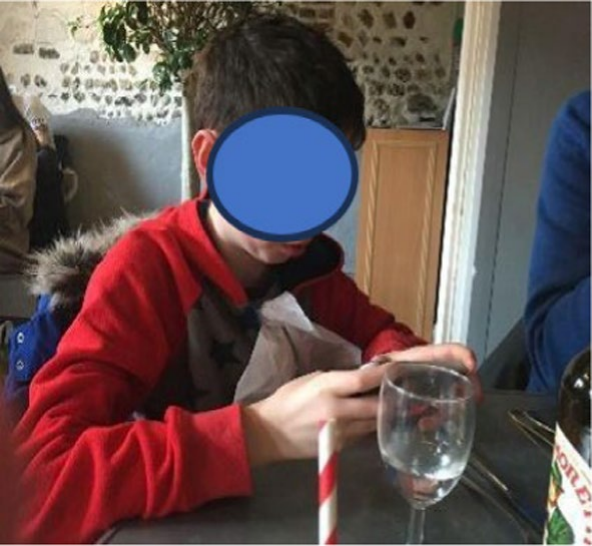

#### 3.3.1 Group experiential theme 1: “There is fun… and there is tough stuff too sometimes”: relationship between twins

This theme captures the relationship between the twins which is characterised by numerous intimate interactions. The twins spoke of their enjoyment from playing together, experiencing conflict during play and the need to adapt their routines to accommodate their twin. Their accounts demonstrate a close bond and high levels of empathy for neurodivergent twin.

##### 3.3.1.1 Enjoyment from being together

Most of the twins reported positively on spending time together and the enjoyment and happiness they get from it. Orla adores her brother and playing together makes her very happy.

*“I remember a time in the summer where me and Martha and Max were all outside playing together in the playhouse and we were all having lots of fun…I’ve never felt as happy.”* (Orla)

Reya spoke of the change and improvement of their relationship over time.

*“When we were little, he did not always like understand me but now he does and I can play with him, just like I play with any of my friends.”* (Reya)

##### 3.3.1.2 Intensity of bond between twins

Albeit to varying extents, most of the twins commented on the closeness of their relationship with their twin, their desire to be together and to protect them, often showing extremely good empathy. Some participants such as Reya explicitly referred to their bond, “*yeah I play with him more than anyone in my family, probably*.”

Protecting their twin was noted by four of the children as a main strategy to stay close and show care. Orla stated that she would often feel awkward doing things that her brother could not do so would sit out of them with him.

*“I do not want Max to feel sad, cause if he’s watching other people do things that he cannot do I cannot imagine how it would feel.”* (Orla)

##### 3.3.1.3 Sibling conflict

Throughout the interview, all the twins commented on the struggles within their relationship. Three siblings expressed displeasure with the reduced time together with their twin, due to different interests or their twin’s desire to spend time alone.

*“I feel a bit sad sometimes because I really want to play with him because he’s really fun*.” (Liam)

Amelia expressed her frustrations at her brother not sharing with her: “s*o if Teddy wants the computer and it’s my go, it’s like he just wants it to himself and he just will not let me have a go.”*

Two of the twins commented on feeling that they did not have a twin, it was more like having a younger sibling.

*“I would not say the same age, as in I feel he’s a bit younger*.” (Reya)

#### 3.3.2 Group experiential theme 2: “…I get it, I know him so well.” Neurotypical twins as experts of their brothers’ neurodivergence

Albeit it to varying extents, all the neurotypical twins discussed their understanding of their brother’s condition and the impact it has on their relationship. At its core, this theme demonstrated that the twin’s neurodivergence has had an impact on the neurodivergent twin but that they understand their brother’s idiosyncratic behaviour.

##### 3.3.2.1 Perception of condition and behaviour

The children demonstrated an awareness of their twin’s neurodivergence, attributing this understanding to their observations of behaviour and/or information primarily provided by their mothers. The majority of the twins concentrated on characteristics and behaviours that could be viewed as difficult or different and occasionally concerning. Liam commented on the mood swings they observed stating, “*he gets very angry and silly*.” Orla and Amelia both referred to the ramifications of their twin’s neurodivergence.

Siblings also were able to discriminate between behaviours of concern and behaviours that just happen.

*“Sometimes he accidentally shoots his hands up and hurts me. Sometimes he does mean to hurt me because he’s trying to be silly but sometimes he does not*.” (Orla)

All siblings also demonstrated good knowledge of situations that can upset their twin.

Amelia spoke of her understanding of what affects her brother, “*all sorts of high pitched noises, really loud noises…a musical festival, he would not like that*.”

##### 3.3.2.2 Adapting and change

Three of the children discussed how they have to adapt their relationship and how they do not want to change it. Emmanuel pointed out that he would not like to change the way his brother is “*I like the way he is now*.”

Amelia discussed how she has to adapt to the mood of her brother before deciding whether she can play with him or not.

*“Yeah, sometimes I can (play with him) but sometimes I cannot…if he’s in a good mood or a bad mood.”* (Amelia)

#### 3.3.3 Group experiential theme 3: “It’s fun to be a twin but sometimes I feel worried.” Neurotypical twins’ perceived struggles and needs

This theme includes descriptions of the neurotypical twins’ perceived struggles and needs. It shows the difficulties associated with having a twin with a neurodivergence which for the majority of the twins led to worries about them. Their descriptions show that the support they receive varies too.

##### 3.3.3.1 Current worries/difficulties

Most of the twins discussed the difficulties surrounding their twin’s neurodivergence, for instance, restrictions on leisure activities, having friends round to play and trying to do homework around their twin. Reya commented that “*I’d like to go to more places as in some places I’m not allowed to go, in case Ibrahim breaks a rule or something*.”

A lot of the worries the twins had were sounding the health of their twin and thinking about their future. Joshua said, “*when he has seizures I do not like it*.” Reya elaborated on how often she feels worried about the future “*I worry about how later when I have a job…who will look after him.”*

##### 3.3.3.2 Perceived support

The children stated that they receive support from several different networks. Two of the children stated that they get support from their families. Emmanuel spoke about his mother, “*she’s always there*.” Reya talked about getting support from her brother “*I talk to him quite a bit, to be honest.”*

Some stated that they have supportive friends whereas others stated that their friends do not understand. Amelia commented on making new friends at community support groups she said she had “*friends there she could talk to*.”

#### 3.3.4 Group experiential theme 4: “My mum spends a lot of time with him, helping him.” Neurotypical twins’ relationship with parents

This theme relates to the relationship the neurotypical twin has with their parents. In the main, this theme shows that the neurotypical twin believes that their parents treat them differently but at the same time they were also concerned about the health of their parents.

##### 3.3.4.1 Differentiated parenting

This captures the neurotypical twins’ beliefs that there are different rules and expectations for each of the twins and the unfairness of this. Liam commented on feeling “*a bit jealous*” of how much his brother is allowed to use his phone compared to him.

##### 3.3.4.2 Worries about parental health

Two of the children, Orla and Amelia showed concerns about their mothers, the extra work they had to do and whether they were looking after themselves enough.

*“I feel a bit bad for mummy some days because sometimes Max wants to eat her dinner instead of his but mummy does not really get to have much food because she needs to help Max with his”* (Orla)

##### 3.3.4.3 IPA themes for participating mothers

Analysis of the seven transcripts from the mother’s interviews resulted in the emergence of four group experiential themes, each of which included a list of personal experiential themes (see Table 3).

**Table 3:** Mothers’ group experiential themes, personal experiential themes and example photos from interviews.

Group experiential theme	Personal experiential theme	Example photo
“They’ve got just a kind of an innate sense of what each other wants, they are kind of really on each other’s wavelength.” Mothers’ perception of the relationship between twins	Twin closeness Lack of twin closeness Expectations of the twin relationship	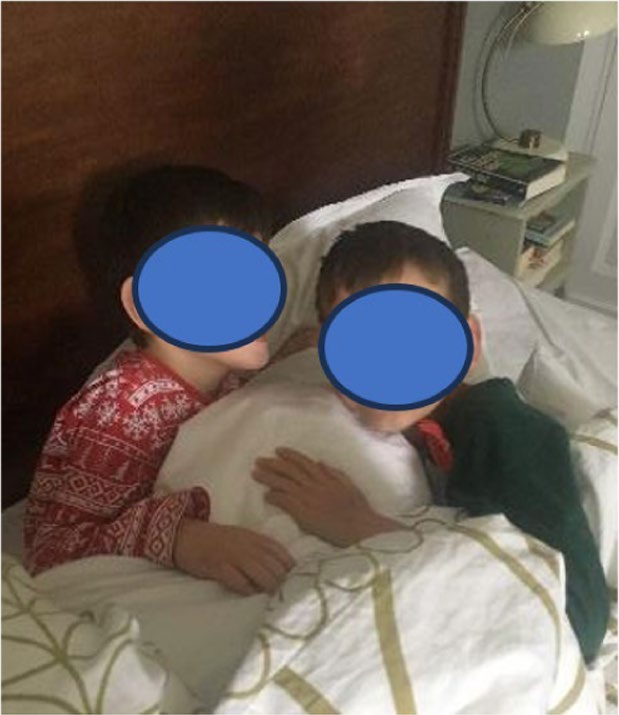
“She knows as much as there is, as much as I know.” Mothers’ perception of the neurotypical twin as an expert of their brother’s condition	Neurotypical twins’ perception of brothers’ condition and behaviour Emotional/ behavioural impact	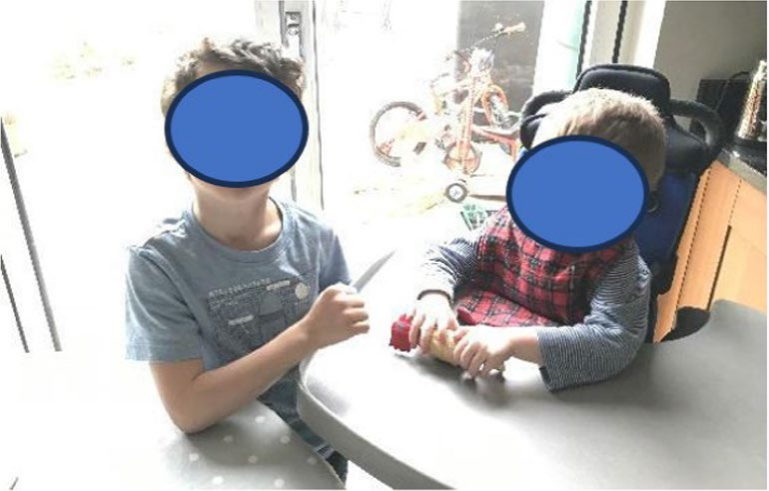
“It restricts a lot of her life, compared to her peers.” Neurotypical twins’ perceived struggles and needs	Current worries/difficulties Perceived support	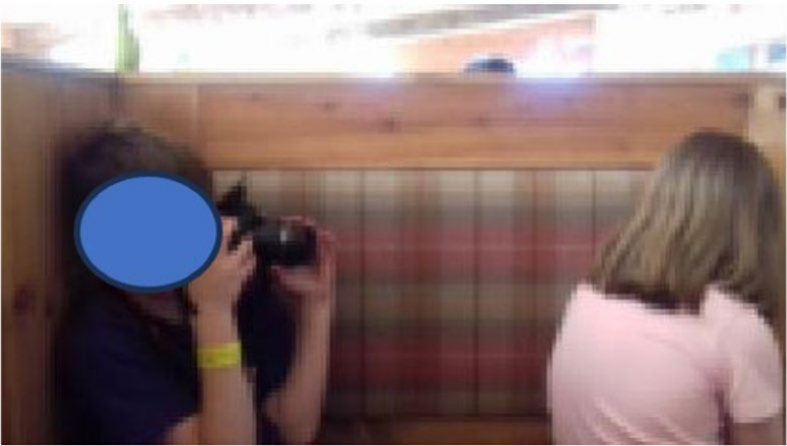
“There’s a few things we can enjoy (altogether) as a family but it’s very hard.” Family experiences	Negotiating family time	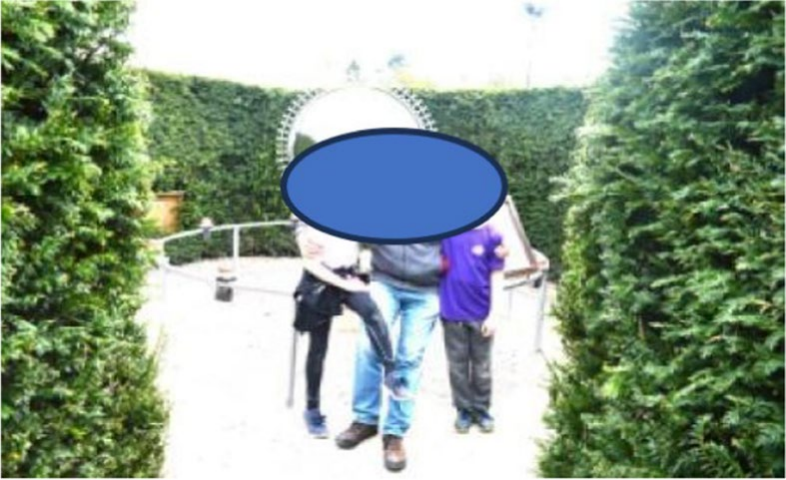

#### 3.3.5 Group experiential theme 1: “They’ve got just a kind of an innate sense of what each other wants, they are kind of really on each other’s wavelength.” Mothers’ perception of the relationship between twins

This theme captures the relationship the mothers perceive the twins to have. It includes the personal experiential themes of the closeness and lack of closeness between the twins, the expectations of the relationship twins should have and the connection between them. This theme illustrates that, while the twins shared a strong bond, their distinct personalities and individual needs, as recognised by their mothers, contributed to a gap that introduced some emotional distance between them.

##### 3.3.5.1 Twin closeness

Albeit to varying extents, all the mothers discussed the closeness between the twins. Jill discussed how Max makes everything better for Orla “*he’s the first person that she goes to when she feels sort of a bit lost or whatever*.” Anne commented that they have such a close relationship that Emmanuel understands Patrick more than her, “Emmanuel gets Patrick, he understands sometimes even when I do not.” Ameena spoke about Reya’s acceptance of her brother’s condition.

“She’s accepting of it, because she’s never know different because she does not have a comparison, she does not know life without autism.” (Ameena)

##### 3.3.5.2 Lack of twin closeness

While all mothers recognised reciprocity in twins’ day-to-day exchanges, three mothers commented on how it often felt they lived parallel lives due to atypical responses or needs expressed by neurodivergent twin. Kelly spoke of them “playing together but they are not together” and Connie pointed out that Joshua is “in some ways an only child” due to the many times he had to take own initiatives and be creative on his own with toys.

Julia remarked that Amelia and Teddy have always been different, and they require very different type of help or attention.

*“Right from the beginning, they both had very different needs and very different personalities*.” (Julia)

Connie wondered how often her neurotypical son “wishes his twin was more like him so they could play together” as his frequent falls when he walks or his night-time seizures often scare him.

##### 3.3.5.3 Expectations of the twin relationship

Throughout each interview, the mothers discussed their twin’s relationships in comparison to what they expected a twin relationship to be like. Reya admitted she had no examples of how twins typically interact and she was not sure what to expect from her sons. She always thought that typical twins are closer compared to her twin sons. Julia did not believe her twins’ relationship was profoundly different but that “*the challenges are exacerbated by the fact that Teddy is not neurotypical and is autistic*.” Connie remarked that she had never seen her twins have a “particular twin bond.”

*“It was kind of easier to think of them as a kind of an older brother and a younger sibling really.”* (Connie)

#### 3.3.6 Group experiential theme 2: “She knows as much as there is, as much as I know.” Mothers’ perception of the neurotypical twin as an expert of their brother’s condition

This theme includes the mother’s perception of the impact the brother’s condition has on the neurotypical twin. It captures how the neurotypical twin does have a good understanding of their brother’s condition, especially for their age. It also shows the behavioural and emotional impact of their brother’s condition on the twin, in terms of coping and parental expectations.

##### 3.3.6.1 Neurotypical twins’ perception of their brother’s condition and behaviour

All the mothers discussed their neurotypical twins’ understanding of their brother’s condition. In the main, the TD twins have been told about their twin brother’s condition. Julia commented that they tried to explain it all but some of it is hard to understand.

*“We’ve tried to explain it as much as possible, but I think it’s a really hard thing to understand and particularly some of the more anti-social stuff like the faecal smearing.”* (Julia)

Ameena mentioned that she thinks that Reya understands her brother’s condition in terms of the “*impact it makes on family and on her*.”

##### 3.3.6.2 Emotional/behavioural impact

Most of the mothers commented on the emotional and behavioural impact of the twin’s condition on their TD twin. Louise showed concerns about Elena’s behaviour, “*I’m really conscious that maybe a lot of her behavioural stuff is attention*.” Some participants, such as Ameena, explicitly referred to coping, she questioned whether Reya copes with her twin’s condition “*because she has to cope*.”

Julia talked about the expectations she has on Amelia with regards to Teddy.

*“So it’s always on Teddy’s terms and sometimes I unrealistically and unreasonably expect Amelia just to fall in with Teddy’s terms just in order to make life easier for all of us…”* (Julia)

#### 3.3.7 Group experiential theme 3: “It restricts a lot of her life, compared to her peers.” Neurotypical twins’ perceived struggles and needs

This theme relates to the struggles and needs of the neurotypical twin and the support they receive to help with these. In the main, this theme shows that there are difficulties associated with having a twin brother with a neurodivergence but there is some support out there to help them.

##### 3.3.7.1 Current worries/difficulties

This captures the worries and difficulties the mothers perceived their neurotypical twin to be experiencing. The difficulties include restrictions, and less freedom compared to friends. Kelly noted that Joshua “*gets less freedom…because it’s difficult to say he can do something if Jonny cannot*.” Ameena commented that Reya has more restrictions compared to her friends “*it limits her in what she can do*.”

In terms of worries a lot of the neurotypical twins worried about their twin’s health and the future. Jill remarked that Orla suffered from guilt, asking “*why it was Max and not me*.”

##### 3.3.7.2 Perceived support

The mother’s feelings around support for their neurotypical twin were very mixed. Some mothers, for example, Anne, commented: *“I’ve really struggled to get that support*.” Kelly noted that she had not looked for support, but she did not “*get the impression there is*” any support.

On the other hand, Julia remarked that they have found very good support.

*“We are very lucky because what we have done is we have found people who can help and support us.”* (Julia)

#### 3.3.8 Group experiential theme 4: “There’s a few things we can enjoy as a family but it’s very hard.” Family experiences

This theme shows the impact of the twin’s neurodivergence on them as a family unit. It showed that the families generally struggle to be able to do things together, which results in dividing the family to do separate activities.

##### 3.3.8.1 Negotiating family time

This shows the difficulties associated with family life when bringing up a twin with a condition. Connie commented that “*it’s very rare that we do, that we can find things to do together as a shared experience*.” This inability to do things as a family has often resulted in families doing separate activities, as noted by Julia “*I think we have probably done too much of this now………we have done a lot of separate activities*.”

Jill, on the other hand, has been able to find lots of activities for disabled children and their families which “*benefits all of us to go and do that together*.”

## 4 Discussion

The current study explored the experiences and perceived needs of neurotypical twins who have a neurodivergent co-twin. The perspectives of both the neurotypical twin and their mother were analysed. The use of photo-elicitation interviews provided the neurotypical twins and their mothers with the opportunity to talk about the most important aspects of their experiences without imposing a set of *a priori*, fixed questions.

These findings offer important insights into the experiences and perceptions of twin sibling relationships. These are novel and expand previous literature on twin sibling relationships from multiple perspectives. In particular, they shed further light on the under-researched relationships between neurotypical and neurodivergent twins by seeking the first-person qualitative accounts of both neurotypical twins and their mothers.

Previous sibling studies have found conflicting results, some suggesting that growing up with a sibling with a disability has negative effects on the neurotypical sibling (Verte et al., 2003) whereas others have found more positive effects (Kaminsky and Dewey, 2001). Our findings report that growing up with a neurodivergent twin can have both positive and challenging effects on a personal, sibling and whole-family level. Group Experiential Theme 1 reflects this and discusses the relationship between the twins. The story told through this theme endorses the commonly believed stereotype of a close bond between twins (Bekkhus et al., 2014). The neurotypical twins and their mothers both spoke of the enjoyment the neurotypical twins get from being with their twin, their desire to protect their twin and their understanding of each other. These findings are in line with previous research on twin sibling relationships which reports that, overall, siblings enjoy spending time together (Sage and Jegatheesan, 2010) and makes explicit references to feelings of love and warmth (Petalas et al., 2009; Rivers and Stoneman, 2003). Our findings challenge the still-prevalent stigmatising and deficit-based narratives that view neurodivergence as a deficit that solely has a negative impact on family dynamics (Kapp et al., 2013; Karst and Van Hecke, 2012). Different forms of neurodivergence, such as autism, are still stereotypically portrayed in news and other media (Mittmann et al., 2024).

The positive reflections of their relationship appeared to coexist with difficulties which both the neurotypical twins and mothers described such as conflict, living parallel lives and being seen as an older sibling and younger brother. To our knowledge, this is the first time that twin relationships in childhood, in which one twin is neurodivergent, have been explored. This opens the way for future research to further examine the dynamics between family members in families with twins and triplets with different neurotypes. Conflict amongst siblings was discussed by the neurotypical twin and mirrored the findings from Petalas et al. (2013) who found that neurodivergent siblings did not want to socialise as much as or in similar ways with the neurotypical siblings. This was a complaint from some of the twins in this study who enjoyed their twin sibling’s company and desired to spend more time with them.

The accounts from the neurotypical twins found that they did not wish to change their twin; contrariwise, some of them would like to spend more time together, but they showed an acceptance of their situation and ability to adapt to their twin’s needs. This is in line with Gorjy et al. (2017) who stated that once siblings recognised that their sibling is different and always will be, they see their situation as normal. It was found that the neurodivergent co-twin is embedded in the neurotypical twin’s siblinghood experiences, for example, one of the mothers in the study commented that “she’s never known different because she does not have a comparison, she does not know life without autism.” However, some mothers worried about potential lack of closeness due to differences that define each pair of twins and assumed that the neurotypical twin may feel distress or lonely.

The accounts in Group Experiential Theme 2 discussed the neurotypical twin’s perceptions and understanding of their brother’s neurodivergence. All neurotypical twins had a relatively good understanding of their brother’s neurodivergence and its impact on the family. A good understanding between siblings can lead to acceptance and empathy (Mascha and Boucher, 2006; Ünal and Baran, 2011). Glasberg (2000) suggested that a good understanding of the neurodivergence should protect the neurotypical sibling from unwarranted fears and worries. This was not found in this study, as most of the neurotypical twins and their mothers commented on the neurotypical twin’s worries about their neurodivergent twin. These worries included concerns about their health and their long-term future. This is in line with previous research showing that siblings of neurodivergent children (e.g., autistic) may experience increased levels of anxiety and stress (Quatrosi et al., 2023). Future research with larger sample sizes could further elucidate the emotional and psychological impact of twin relationships on both neurotypical and neurodivergent siblings.

As seen in Group Experiential Theme 3 most of the neurotypical twins and mothers discussed the sources of support they had or the struggles they have had to get the support needed. The children detailed the support they received from their families and how their friends showed a lack of understanding which is in line with previous studies (Petalas et al., 2009; Barr and McLeod, 2010; Stampoltzis et al., 2014). A lack of support could lead to adverse effects on the neurotypical sibling. Gold (1993) reported higher levels of self-reported depression in siblings with a brother with a neurodivergence, however, this was not measured in this study. Some promising findings show that sibling support groups may help the neurotypical twins to share their experiences, talk with siblings in similar situations and develop a supportive peer network (Jones et al., 2020). The twins in this study who attended support groups reported they felt they had someone to talk to when joining these support groups, however, not everyone was able to access the necessary support and one of the mothers felt it was not necessary yet.

The twins discussed the restrictions they faced on leisure activities and not being able to do the same activities or have the same freedom as their friends, which was also found by Petalas et al. (2009). However, there were no complaints about family plans being disrupted as found by Barr and McLeod (2010) and no resentment was felt by the neurotypical twin’s contrary to findings by Kendall (1999). This may be an example of the twins not knowing a different way of living; they may be able to see the different lives their friends have but they have never experienced this so do not see their sibling’s neurodivergence as a restriction. The mothers, on the other hand, reported how it restricts their neurotypical children’s lives, often creating worries and challenges.

Our findings highlight the fundamental need to create supportive social systems with siblings and their relationships at the heart of them. They underline the significance of actively involving disabled children, their siblings and parents/carers in the co-design of holistic, family support schemes to ensure these are in line with their identified priorities and areas of need. Schools can also be valuable sources of support for siblings. For example, recent research reports that siblings appreciate having the opportunity to speak with a trusted school staff member about their home life, being understood when struggling with homework and deadlines and having access to a quiet, safe space in school to decompress and find their flow (Kassa and Pavlopoulou, 2024).

A long-held assumption is that siblings of children with a disability have significantly more adjustment, emotional and conduction problems (Griffith et al., 2014). The majority of these studies used the mothers to rate the problems their children had. This study sought the perspectives of both the mothers and the neurotypical twins. We found a lot of commonalities in the themes between the mothers and the neurotypical twins, which contradicts the results found by Hastings and Petalas (2014). A key difference in the accounts of mothers was that they were more worried about twins’ differences and conflicts.

Finally, our findings suggest that what is often described in the literature as parentification may emerge very early, with our youngest participant being just 5 years old. These early caregiving dynamics reflect both a labour of love and a potential source of stress, shaping prosocial behaviours while placing emotional demands on the typically developing twin. This vertical, caregiving-oriented relationship echoes sibling dynamics more typical of different-age siblings than same-age twin dyads (East, 2010; Aldridge and Becker, 1999; Cree, 2003). These caregiving dynamics—where neurotypical twins take on protective or supportive roles—often mirror those more commonly observed in mixed-age sibling relationships.

**Figure 1:**
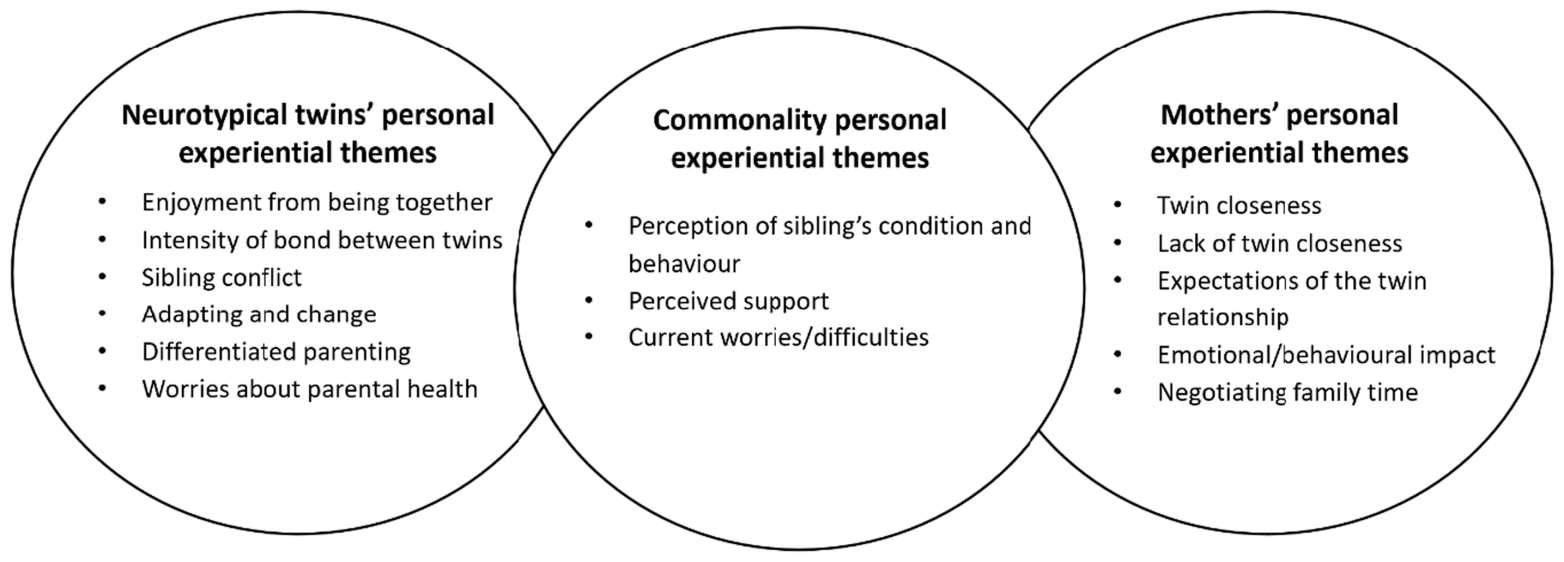
Commonality and different themes between neurotypical twins and their mothers.

Figure 1 shows that three themes were common among neurotypical siblings and their mothers, indicating that both groups share relatively similar understandings of twin sibling relationships.

### 4.1 Methodological strengths and limitations

Currently, there is a dearth of research around neurotypical twins’ experiences and perceived needs when they have a neurodivergent co-twin. This study offers valuable insights into the relationships between neurotypical and neurodivergent twins that have been consistently overlooked. A significant strength is that this study sheds further light on the multi-dimensional perspectives of twin sibling relationships in childhood by drawing directly on the voices of neurotypical twins and their mothers.

In addition, a lot of previous research has only used mothers’ ratings and opinions of how the child is feeling (Griffith et al., 2014; Meyer et al., 2011; Petalas et al., 2012). This study used a multi-informant approach, gathering first-hand perspectives from neurotypical twins as well as their mothers, which enabled us to gain a detailed understanding of their experiences—highlighting both positive aspects and challenges. Furthermore, the use of participant-led creative methods such as photo-elicitation was a valuable and rich tool that allowed us to reflect on the day-to-day experiences of families, focusing on the themes participants themselves identified as important.

There are limitations to this study. First, the sample size is small but acceptable for a phenomenological study (Smith et al., 2009). One mother also participated without their neurotypical child, which is a limitation. This may explain, to some extent, the inconsistency between our study’s findings and those of previous studies. Second, the photos were taken over a short period of time. This is a limitation as the study may have failed to capture key life experiences across various time points that may impact twins’ relationships and family dynamics in different ways (e.g., transition school periods, holidays). Future research should collect more detailed information from participants about the specific timing of photo-taking to better understand their decisions behind each photo selection. Importantly, this study did not include the voices of the neurodivergent twins. Including their perspectives in future research could offer a more holistic understanding of the sibling relationship, highlighting how both siblings perceive and experience their bond, and how they navigate everyday joys and challenges.

Third, in this study, we chose to seek only the mothers’ perspectives as these are more often involved in parenting based on existing research, and thus, they are more likely to have a more holistic understanding of siblings’ relationships (Zahl et al., 2024). However, this is a limitation. Mothers and fathers may experience a different point of view with regards to their neurotypical twin’s experiences of growing up with a neurodivergent co-twin. Although fathers’ perspectives are generally under-explored, fathers’ contributions are central for children’s wellbeing and during intervention efforts (Rankin et al., 2019). Fourth, although we made considerable efforts to create a friendly and nonjudgmental environment for participants to express their ideas, personal biases, such as social desirability bias, may have influenced what everyday experiences they chose to share.

Fifth, another limitation is the representativeness of the sample, all the mothers apart from one were white and married, so they all came from a similar cultural background. In addition, all the neurodivergent twins were male, so the relationships between these siblings may not be the same as between twin sisters for example. Finally, the participant twins in our study reported opportunities to develop double empathy (Milton, 2012), they understood each other and normalised differences and conflicts despite their different and unique life experiences. Understanding this has important implications for siblings’ researchers as well as practitioners and therapists who provide family-based interventions. These interventions should place whole family relationships at the core and explore dynamics within neuro-mixed families with a sense of curiosity, empathy and understanding, moving away from traditional medical models that view neurodivergence as something that needs to be “fixed” to promote family wellbeing. Future research should further explore how twins’ intersected identities can impact their relationship, for example, by including more diverse samples in terms of socioeconomic status and ethnicity. Sixth, there is a lack of longitudinal studies on neurotypical and neurodivergent twins/triplets. It may be valuable to look at the impact of twins’ experiences and perceptions over time. This would enable us to determine whether siblings’ relationships remain positive with increasing age and whether there is any impact on the neurotypical twins in the long term.

## 5 Conclusion

All too often, children of multiple births are thought of as a singular unit, instead of being recognised as two distinct individuals. There is a lack of understanding regarding the individuality and relationships of twins during their upbringing. Additionally, we are unaware of how parents view their twins’ relationship and how they nurture independence while addressing each twin’s unique emotional and developmental requirements, particularly when one twin is born with a disability. To our knowledge, this study is one of the first to be conducted on twins with a neurodivergent co-twin by eliciting the first-person qualitative accounts of neurotypical twins and their mothers. Overall, our findings highlight the joy and close bond that twin siblings get from each other and their strong understanding of their co-twin. They also reveal the vulnerabilities of the neurotypical twins and the worries and struggles they face. Our findings highlight several areas that may be beneficial during post-diagnostic support, particularly in providing practical, and emotional guidance on how to communicate information about behaviour and disabilities to twins. Our findings can also inform future research to determine whether they can be generalised to neurodivergent youth twin populations. Further research is needed to identify elements of psychosocial support needed for these families and to influence policy and practice.

## Data Availability

The datasets presented in this article are not readily available to protect the anonymity of participants. Questions regarding the datasets should be directed to the corresponding author.
