# Supplementary material for: The experiences, perspectives and needs of families raising neurodivergent twins: a multi-informant photo-elicitation exploratory study
Source: Front Psychol. 2025 Jun 3;16:1571108. doi: 10.3389/fpsyg.2025.1571108 (PMC12175008; doi:10.3389/fpsyg.2025.1571108)
Supplement: Supplementary file 1 [file Table_1.docx]

**Supplementary Material**

**Supplementary Material 1: Co-Produced Survey Questions for Families**

How can research help twins, triplets, multiple births with a health condition and/ or disability (such as autism, epilepsy etc)?

Question 1. Sibling relationships

- Vitally important
- Fairly important
- Not important enough
- I don’t/feel unsure

Question 2. Sleep problems, sleep support

- Vitally important
- Fairly important
- Not important enough
- I don’t/feel unsure

Question 3. Social stigma, social support

- Vitally important
- Fairly important
- Not important enough
- I don’t/feel unsure

Question 4. School provision, homework, after-school activities

- Vitally important
- Fairly important
- Not important enough
- I don’t/feel unsure

Question 5. Martial relationships

- Vitally important
- Fairly important
- Not important enough
- I don’t/feel unsure

Question 6. Please use this box to outline ONE question you would like researched, about families with twins/triplets/multiple births

Question 7. Please use this box to outline the SECOND question you would like researched, about families with twins/triplets/multiple births

Question 8. So that we know broadly the range of families who have helped us we would be grateful if you could provide us with some information about you. What is your gender?

- Female
- Male
- Prefer not to say
- Non-binary/other

Question 9. Please add your full name.

Question 10. Please add your contact details below.

Name___________________________________________________________

Company_____ __________________________________________________

Address_________________________________________________________

Address 2________________________________________________________

City/Town________________________________________________________

State/Province____________________________________________________

ZIP/Postal Code___________________________________________________

Country__________________________________________________________

Email address ____________________________________________________

Phone number ____________________________________________________

Question 11. Please tell us a little bit about your twins/triplets/multiple births. Provide us with age, gender, diagnosis, full-term or preterm details for each child. You may also add anything else you would like to add.

Question 12. Do you have any other comments, questions, or concerns?

**Supplementary Material 2: Co-Produced Questionnaire for Mothers**

**Mothers' experiences of raising twins where one twin has a neurodevelopmental**

**condition**

Welcome to the research study!  
 
We are interested in understanding mothers' experiences of raising a child with a neurodevelopmental condition. To gather this information, I will be asking you to complete an online questionnaire containing questions on the following:

·    Background information (for example the names and ages of your family members)

·    The diagnosis of the child (for example your perceptions of your child’s symptoms).

·    The quality of life of the parents.

For the purpose of this study, a 'typically developing' person, is someone who has the behaviour, intellectual ability and functional skills typically seen in children of their age.  

The questionnaire should take you around 20 minutes to complete. Your participation in this research is voluntary. You have the right to withdraw at any point during the study, for any reason, and without any prejudice. 
 
All responses are anonymous and confidential. You will be identified by a unique number and the data will be used for research purposes only. Once the research is complete and the results and recommendations are established you will be provided with a summary of the results.
  
 If you would like to contact the researcher in the study to discuss this questionnaire, please e-mail [researcher’s email].  Please note, you will only be able to access the questionnaire once you have completed the informed consent section and all the tick boxes have been ticked. 
This survey will be best displayed on a laptop or desktop computer.  Some features may be less compatible for use on a mobile device. 

If you consent to taking part in this questionnaire please confirm so by selecting all of the boxes below and signing your name and the date.

- I confirm that I have read and understood the information sheet, and have had the opportunity to consider the information, ask questions, and have had these questions adequately answered.
- I understand that my participation is voluntary and that I am free to withdraw at any time, without giving any reason.
- I know that I can refuse to answer any or all of the questions and that I can withdraw from the interview at any point.
- I understand that the questionnaires will be kept secure and destroyed at the end of the project. I know that all data will be kept under the terms of the General Data Protection Regulation (GDPR).
- I agree that small direct quotes may be used in reports (these will be anoymised).
- I understand that in exceptional circumstances anonymity and confidentiality would have to be broken, for example, if it was felt that practice was putting children at risk, or there were concerns regarding professional misconduct. In these circumstances, advice would be sought from a senior manager from another local authority who will advise us as to the appropriate course of action and as to whether we need to inform the authority of what you have told us.
- I consent to taking part in this study.

I consent to participating in this research.

- Name ________________________________________________
- Date _______________________________________________

The first section contains questions about the demographics of your family and your children.

Please list the names, ages and gender of all the people who live in your family household.

|  | Name | Age | Gender | Please indicate the child/children with a neurodevelopmental condition |
| --- | --- | --- | --- | --- |
| Person 1 |  |  |  |  |
| Person 2 |  |  |  |  |
| Person 3 |  |  |  |  |
| Person 4 |  |  |  |  |
| Person 5 |  |  |  |  |
| Person 6 |  |  |  |  |
| Person 7 |  |  |  |  |
| Person 8 |  |  |  |  |

Question 2. How would you describe your twins/triplets?

- Identical
- Non-identical
- Mixed triplets

Question 2a. Is the child/children with the neurodevelopmental condition(s)

- Identical
- Non-identical

Question 3. What is your marital status?

- Married
- Living together
- Divorced
- Separated
- Single/never married
- Widowed

Question 4. What is your ethnicity?

- White
- Black/African/Caribbean/Black British
- Asian/British Asian
- Mixed/multiple ethnic groups
- Other Ethnic group

Question 5. What is the primary diagnosis of your child/children with the neurodevelopmental condition(s)?

Question 6. At what age did your child/children with the neurodevelopmental condition(s) receive the diagnosis?

Question 7. Is your child/children with the neurodevelopmental condition(s) receiving medication or counselling?

Question 8. Has either parent been diagnosed with a neurodevelopmental condition?

- Yes
- No

Question 8a. Please provide details of the parents diagnosis

Question 9. Is your typically developing twin/triplets aware of their twin/triplets siblings diagnosis?

- Yes
- No

Question 9a. How much information does the typically developing twin/triplets know about their twin/triplets siblings diagnosis?

Question 10. Does your 'typically developing' child have any current disability or psychiatric diagnoses?

- Yes
- No

Question 10a Please provide details of the diagnoses.

This section includes questions which look at the relationship you have with your child/children with the neurodevelopmental condition, your understanding of their behaviour and your views about the future. Please select the option which indicates how you feel.

Question 11. I feel I understand the behaviour of my child/children with the neurodevelopmental condition.

- Strongly agree
- Agree
- Unsure
- Disagree
- Strongly disagree
- Not applicable

Question 12. I do not understand the diagnosis/difficulties of my child/children with the neurodevelopmental condition.

- Strongly agree
- Agree
- Unsure
- Disagree
- Strongly disagree
- Not applicable

Question 13. I am confident about managing the behaviour of my child/children with the neurodevelopmental condition.

- Strongly agree
- Agree
- Unsure
- Disagree
- Strongly disagree
- Not applicable

Question 14. I feel I am failing as a parent.

- Strongly agree
- Agree
- Unsure
- Disagree
- Strongly disagree
- Not applicable

Question 15. I have times when I am able to feel close to my child/children with the neurodevelopmental condition.

- Strongly agree
- Agree
- Unsure
- Disagree
- Strongly disagree
- Not applicable

Question 16. I feel more hopeful about the future.

- Strongly agree
- Agree
- Unsure
- Disagree
- Strongly disagree
- Not applicable

Question 17. I do not feel we are coping as a family

- Strongly agree
- Agree
- Unsure
- Disagree
- Strongly disagree
- Not applicable

1. This section contains questions about your quality of life. Please select the option which indicates how you feel.

Question 18. How would you rate your quality of life?

- Very poor
- Poor
- Neither poor nor good
- Good
- Very good

Question 19. How satisfied are you with your health?

- Very dissatisfied
- Dissatisfied
- Neither satisfied or dissatisfied
- Satisfied
- Very satisfied

|  |  |
| --- | --- |

The following questions ask how much you have experienced certain things in the last two weeks.

Question 20. To what extent do you feel that physical pain prevents you from doing what you need to do?

- Not at all
- A little
- A moderate amount
- Very much
- An extreme amount

Question 21. How much do you need any medical treatment to function in your daily life?

- Not at all
- A little
- A moderate amount
- Very much
- An extreme amount

Question 22. How much do you enjoy life?

- Not at all
- A little
- A moderate amount
- Very much
- An extreme amount

Question 23. To what extent do you feel your life to be meaningful?

- Not at all
- A little
- A moderate amount
- Very much
- An extreme amount

Question 24. How well are you able to concentrate?

- Not at all
- A little
- A moderate amount
- Very much
- Extremely

Question 25. How safe do you feel in your daily life?

- Not at all
- A little
- A moderate amount
- Very much
- Extremely

Question 26. How healthy is your physical environment?

- Not at all
- A little
- A moderate amount
- Very much
- Extremely

The following questions ask about how completely you experience or were able to do certain things in the last two weeks.

Question 27. Do you have enough energy for everyday life?

- Not at all
- A little
- Moderately
- Mostly
- Completely

Question 28. Are you able to accept your bodily appearance?

- Not at all
- A little
- Moderately
- Mostly
- Completely

Question 29. Have you enough money to meet your needs?

- Not at all
- A little
- Moderately
- Mostly
- Completely

Question 30. How available to you is the information that you need in your day-to-day life?

- Not at all
- A little
- Moderately
- Mostly
- Completely

Question 31. To what extent do you have the opportunity for leisure activities?

- Not at all
- A little
- Moderately
- Mostly
- Completely

Question 32. How well are you able to get around?

- Very poor
- Poor
- Neither poor nor good
- Good
- Very good

The following questions ask you to say how good or satisfied you have felt about various aspects of your life over the last two weeks.

Question 33. How satisfied are you with your sleep?

- Very dissatisfied
- Dissatisfied
- Neither satisfied nor dissatisfied
- Satisfied
- Very satisfied

Question 34. How satisfied are you with your ability to perform your daily living activities

- Very dissatisfied
- Dissatisfied
- Neither satisfied nor dissatisfied
- Satisfied
- Very satisfied

Question 35. How satisfied are you with your capacity for work?

- Very dissatisfied
- Dissatisfied
- Neither satisfied nor dissatisfied
- Satisfied
- Very satisfied

Question 36. How satisfied are you with yourself?

- Very dissatisfied
- Dissatisfied
- Neither satisfied nor dissatisfied
- Satisfied
- Very satisfied

Question 37. How satisfied are you with your personal relationships?

- Very dissatisfied
- Dissatisfied
- Neither satisfied nor dissatisfied
- Satisfied
- Very satisfied

Question 38. How satisfied are you with your sex life?

- Very dissatisfied
- Dissatisfied
- Neither satisfied nor dissatisfied
- Satisfied
- Very satisfied

Question 39. How satisfied are you with the support you get from your friends?

- Very dissatisfied
- Dissatisfied
- Neither satisfied nor dissatisfied
- Satisfied
- Very satisfied

Question 40. How satisfied are you with the condition of your living place?

- Very dissatisfied
- Dissatisfied
- Neither satisfied nor dissatisfied
- Satisfied
- Very satisfied

Question 41. How satisfied are you with your access to health services?

- Very dissatisfied
- Dissatisfied
- Neither satisfied nor dissatisfied
- Satisfied
- Very satisfied

Question 42. How satisfied are you with your transport?

- Very dissatisfied
- Dissatisfied
- Neither satisfied nor dissatisfied
- Satisfied
- Very satisfied

The following question refers to how often you have felt or experienced certain things in the last two weeks.

Question 42. How often do you have negative feelings such as blue mood, despair, anxiety, depression?

- Never
- Seldom
- Quiet often
- Very often
- Always

Thank you for taking part in this questionnaire on mother's experiences of raising a child with a neurodevelopmental condition. In particular, the questionnaire focused on the parent-child relationship and the mothers quality of life. We believe that the answers you have given us will help us better understand what it is like to raise twins/triplets where one twin/triplet has a neurodevelopmental condition. 
There were no right or wrong answers, we were genuinely interested in finding out your honest opinion. In the future, the sorts of things you’ve told us could help children like yours and could help parents and other people know what else they can do to make sure their children are getting the right support day to day. Please remember that your responses are confidential and anonymous. You can withdraw your information from the study at any time without giving a reason.  If you want to withdraw or ask any questions about this project my email address is [researcher’s email]. Finally, thank you so much for being involved in this study.

**Supplementary Material 3: Photo-Elicitation Interview Schedules for Children and Mothers**

**Photo Elicitation Interview schedule (child)**

**Introduction**

Initial general conversation to help the participant to feel comfortable and at ease with the researcher.

Outline of the process of the interview, introduce participants to the audio-recording equipment and reiterate that the interview should not take longer than 30 minutes.

Remind the participant that he or she can choose to stop the interview whenever he or she feels like it.

Ensure that interviewee is content to proceed (signs the consent form).

**Icebreakers:**

1. Can you tell me who is in your family?
2. How old is your brother/sister(s)?
3. What are your hobbies? Find out their likes/dislikes/what they are good at etc……
4. What do you know about your brother/sisters condition?

Child will be instructed to select first photograph they wish to talk about.

1. Can you tell me what is happening in this picture?
2. Why did you choose to take this picture?
3. What usually happens before this?
4. What usually happens after this?
5. What else is going on, at the same time, that isn’t in this photo?
6. Tell me about a time when……….
7. Is it always like that? Or is there a time when it wasn’t like that?

Follow-up questions will depend on picture content

1. How does this make you feel?
   1. (if morning) how did this make you feel going to school?
   2. If afternoon/evening – how did this make you feel going to bed?
2. How do you think this made (name) feel? (if relevant)
3. Is there anything you would change about this?
4. Are there other things that are important to you that you didn’t manage to include with your photographs?
5. How have you felt talking about your photographs today?

**General questions:**

1. (if not already answered by photos) Can you describe what a typical day is like for you? (prompts: mornings/after-school, evenings/weekends)
2. Do you feel like there is someone you can talk to?
3. How do you find having a twin brother/sister with a disability?
4. Is there anything I haven’t asked you about (name) that you would like to tell me about?

**Debrief**

**Photo Elicitation Interview schedule (mother)**

**Introduction**

Initial general conversation to help the participant to feel comfortable and at ease with the researcher.

Outline of the process of the interview, introduce participants to the audio-recording equipment and reiterate that the interview should not take longer than 1 hour.

Remind the participant that she can choose to stop the interview whenever he or she feels like it.

Ensure that interviewee is content to proceed (signs the consent form).

**Icebreakers:**

1. Can you tell me about your family?
2. Can you tell me about your twin’s sibling relationship? How do they get along together?
3. What does your typically developing twin know about their brother/sisters condition?

Mother will be instructed to select first photograph they wish to talk about.

1. Can you tell me what is happening in this picture?
2. Why did you choose to take this picture?
3. What usually happens before this?
4. What usually happens after this?
5. What else is going on, at the same time, that isn’t in this photo?
6. Tell me about a time when……….
7. Is it always like that? Or is there a time when it wasn’t like that?

Follow-up questions will depend on picture content

1. How do you think your typically developing twin felt when this picture was taken?
2. Is there anything you would change about this?
3. Are there other things that are important to you that you didn’t manage to include with your photographs?

**General questions:**

1. (if not already answered by photos) Can you describe what a typical day is like for your twin? (prompts: mornings/after-school, evenings/weekends)
2. What do you think are the strengths of their relationship?
3. What do you think your son/daughter finds difficult with having a twin/triplet sibling(s) with a disability?
4. What worries do you think your son/daughter has about their brother/sister?

1. Do you feel like your son/daughter gets the support they need? Is there someone they can talk to?
2. How do you think your son/daughters relationship differs from other typical sibling relationships?
3. Is there anything I haven’t asked you about (name) that you would like to tell me about?

**Debrief**

**Supplementary Material 4: Ethics Forms and Photo-Training Session Materials**

**Please tick as appropriate.**

- - - 1. Have you read the participant information sheet?
      2. Have you had the chance to ask questions and discuss the study?
      3. Have you received enough information about the study?
      4. Do you understand that participation is voluntary and that you are free to withdraw at any time, without giving any reason?
      5. Do you understand that you can refuse to answer any or all of the questions and can withdraw from the interview at any point?
      6. Do you agree to the interview being audio recorded?
      7. Do you give me permission for the things that you say in the interview to be used in reports of the research**,** knowing that your name will not be used?

Please tick one of the boxes below:

- I agree to take part in the research.

OR

- I do not wish to take part in the research, and I have not signed the assent

below.

Please write your initials here if you do not wish to take part:

______________ (initialled by child).

If you agree to take part in the research, please circle which one(s) you agree to below:

1) I would like to have a photo interview

2) The photos can be kept digitally and used as examples in the dissertation

3) The photos can be kept digitally and used to present in future work (e.g conferences)

4) The photos can be kept digitally and used with/without blur (cross out where appropriate)

OR

5) I would like to have an interview without photos

Name ……………………………………………… Signature ……………………………………….. Date ……………………………

Name of researcher ……………………………. Signature ………………………………………… Date ………………………………

**Photo Training Session**

What do I need to do for this research?

You’ll need to take photos over one week to show me your everyday experiences of life as a twin/triplet. They could be things that you enjoy with your brother or sister, things that might be a struggle, what it means to have a twin with a developmental disability..…anything that you think is important to your daily experiences.

**Do**


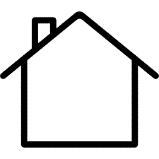

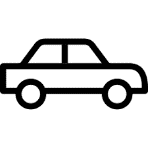

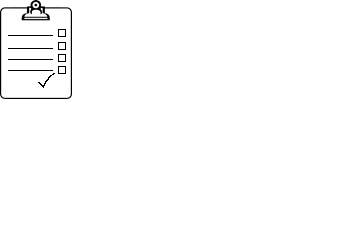

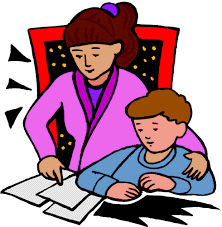


Take photos only at home or in the family car

Check through the photos with your parent/ guardian before the interview session

Get the consent of anybody else who is in a photo (using the mini-consent forms)

Take photos of things that are important to your sibling experience


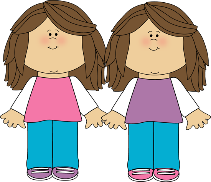


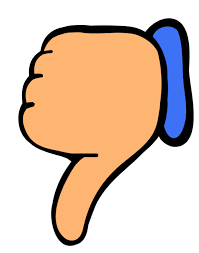
 What do I need to tell the people who I want to be in the photo?

**Don’t**

Take photos of anybody who is under 16 years old

Take photos of anybody who hasn’t given their consent

Take photos of any private body parts or of private activities

Take photos at school, or anywhere that isn’t your home or car

The photo is being taken as part of a research study that you are involved in.

The photo might be used when the results of the study are written up.

If the photo is used, it will be blurred to remove identifying features, but because it is a photo we cannot be certain that they won’t be identified.

If they are happy to be included in this, they will need to tick and sign the mini consent form.

If they don’t want to be involved in the research, make sure that they aren’t included in the photo. If they do sign the consent form, this needs to be handed to me (Vicky) at the interview session.

**Consent Form**

1) I understand that this photo is being taken as part of a research study.

1. I understand that this photo could be shown when the results of the research are written up.
2. I understand that my face and any features that show who I am will be blurred in the photo.
3. However, I understand that because it is a photo it cannot be guaranteed that I will not be able to be identified if the photo is used in the research write-up.

I give my consent to be in this photo (please tick the box and write your initials here):

**Twin Experiences Study**

**Parent/Guardian Checklist**

Before the interview session, please check through the photos your child has taken to make sure they meet the criteria, and that you’re happy for those photos to be used in the interview and the dissertation/future conferences (if you have agreed to this).

Please complete this checklist and bring it to the interview session:

I have reviewed the photos and have removed any photos which are inappropriate.

I have checked that none of the photos fall under the ‘don’t’ category in the criteria.

I am happy for these photos to be used in the interview/dissertation/conferences as previously agreed.

Name (printed):

Thank you!
